# Structure of the type VI secretion system TssK–TssF–TssG baseplate subcomplex revealed by cryo-electron microscopy

**DOI:** 10.1038/s41467-018-07796-5

**Published:** 2018-12-19

**Authors:** Young-Jun Park, Kaitlyn D. Lacourse, Christian Cambillau, Frank DiMaio, Joseph D. Mougous, David Veesler

**Affiliations:** 1 Department of Biochemistry, University of Washington, Seattle, WA 98195 USA; 2 Department of Microbiology, University of Washington, Seattle, WA 98195 USA; 3 Architecture et Fonction des Macromolecules Biologiques, Aix-Marseille Universite, CNRS, Campus de Luminy, Case 932, 13288 Marseille, Cedex 09 France; 4 Howard Hughes Medical Institute, University of Washington, Seattle, WA 98195 USA

## Abstract

Type VI secretion systems (T6SSs) translocate effectors into target cells and are made of a contractile sheath and a tube docked onto a multi-protein transmembrane complex via a baseplate. Although some information is available about the mechanisms of tail contraction leading to effector delivery, the detailed architecture and function of the baseplate remain unknown. Here, we report the 3.7 Å resolution cryo-electron microscopy reconstruction of an enteroaggregative *Escherichia coli* baseplate subcomplex assembled from TssK, TssF and TssG. The structure reveals two TssK trimers interact with a locally pseudo-3-fold symmetrical complex comprising two copies of TssF and one copy of TssG. TssF and TssG are structurally related to each other and to components of the phage T4 baseplate and of the type IV secretion system, strengthening the evolutionary relationships among these macromolecular machines. These results, together with bacterial two-hybrid assays, provide a structural framework to understand the T6SS baseplate architecture.

## Abstract (web summary)

Type VI secretion systems (T6SSs) translocate effector proteins into eukaryotic and bacterial recipient cells and are present in many Gram-negative bacteria. Here the authors present the 3.7 Å cryoEM structure of the *E.coli* T6SS baseplate wedge comprising TssK–TssF–TssG and propose a model for the T6SS baseplate and needle complex.

## Introduction

Bacterial secretion systems transport proteins and nucleic acids across the cell envelope. They serve essential roles in pathogenesis and in enabling communication between cells. Type VI secretion systems (T6SSs) are found in a broad range of Gram-negative bacteria and transport proteins directly into recipient cells^1,2^. They can target either bacterial cells^3^, to provide a selective advantage enabling colonization of specific niches, or eukaryotic cells^4^, to modulate bacteria/host interactions and pathogenesis. As a result, the presence of one or several functional T6SSs correlates with the ability to induce host diseases or disorders for many pathogens^5^.

**Fig. 1:**
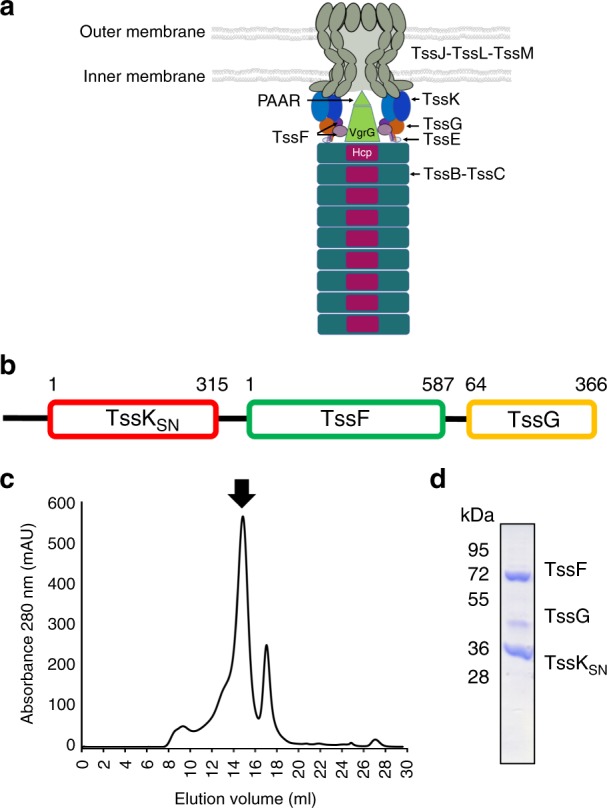
Schematic of the T6SS apparatus, construct design, expression, and purification of the EAEC TssK_SN_-TssF–TssG complex. **a** Schematic of the T6SS apparatus. TssE is rendered semi-transparently owing to uncertainty in its position. TssA is not shown. **b** Schematic representation of the construct used for recombinant expression. **c** Size-exclusion chromatogram after affinity purification. **d** SDS-PAGE of the fraction indicated with an arrow in **c**. Molecular weights from the ladder are indicated on the left. Uncropped gel is shown in Supplementary Figure 3

The T6SS apparatus is assembled from at least 13 distinct types of proteins (Fig. 1a) encoded by a discrete gene cluster, so-called pathogenicity island, which generally also includes a subset of secreted effector and cognate immunity genes^6–8^. The T6SS needle oligomerizes in the bacterial cytoplasm and comprises an inner tube, made of Hcp hexameric rings arranged with helical symmetry, surrounded by TssB–TssC rings forming the sheath. Localization of the needle within a donor cell is mediated by a pre-assembled cytoplasmic baseplate comprising TssK, TssF, TssG, TssE, VgrG, and a PAAR-repeat protein. The baseplate docks to the bacterial envelope via interactions with a complex assembled from the inner membrane proteins TssL and TssM and the outer membrane lipoprotein TssJ.

Contraction of the TssB–TssC sheath is believed to propel the inner tube and VgrG-PAAR spike through the membrane complex of the donor cell, to puncture the envelope of a recipient cell and deliver effectors in the periplasm and/or the cytoplasm^9,10^.

Crystal structures of the Hcp hexamer and of the VgrG trimeric spike revealed part of the building blocks of tailed phages, R-type pyocins, and T6SSs are structurally similar and led to the hypothesis that these nanomachines are evolutionary and functionally related^11–17^. These findings were reinforced upon determination of cryo-electron microscopy (cryoEM) and cryo-electron tomography structures of the TssB–TssC sheath in the contracted and extended states, which provided insights into the mechanism associated with effector delivery to recipient cells and subsequent recycling of the T6SS needle^18–21^. High-resolution crystal structures of isolated domains in combination with low-resolution electron microscopy studies also provided a glimpse of the TssJ–TssL–TssM membrane complex architecture that forms a fivefold symmetrical oligomer spanning the cell envelope^22,23^. This latter complex is evolutionary related to components of the type IVB secretion system (T4BSS) and demonstrates the mosaic origin of the T6SS apparatus^6^.

The lack of high-resolution structural information about the three-dimensional organization of the baseplate complex has hindered our understanding of the architecture and assembly pathway of this key multi-subunit component of the T6SS. TssK was recently shown to resemble lactoccocal phage receptor-binding proteins and to interact with the baseplate complex, via its N-terminal shoulder domain, and with the cytoplasmic domains of TssL and TssM in the membrane complex, through its head domain^24^. Bioinformatics analyses suggested TssE is a baseplate wedge component related to phage T4 gp25, P2 gpW, or Mu Mup46^11,25^. Also, TssF and TssG were proposed to respectively, resemble phage T4 gp6 and gp7, P2 gpJ and gpI, or Mu Mup47 and Mup48^25–27^. TssK is known to form a complex with TssF, and TssG that can be purified endogenously^28^ or upon recombinant overexpression^24,27^ although the stoichiometry, architecture, and role of this complex in the T6SS apparatus remain unclear.

To address this knowledge gap, we report here the 3.7 Å resolution cryoEM structure of the EAEC TssK–TssF–TssG complex. Our results show TssG acts as an adaptor interacting with two TssK trimers and two TssF protomers to form part of a baseplate wedge (which also comprises TssE). We observed that TssF and TssG are structurally related to each other and to components of the phage T4 baseplate and of the T4SS, further strengthening the suggested evolutionary relationships among these macromolecular machines. Integrating our data with previous work, we propose a complete model for the needle and baseplate components and a putative assembly pathway of the T6SS apparatus.

## Results

### Structure determination of TssK–TssF–TssG

**Fig. 2:**
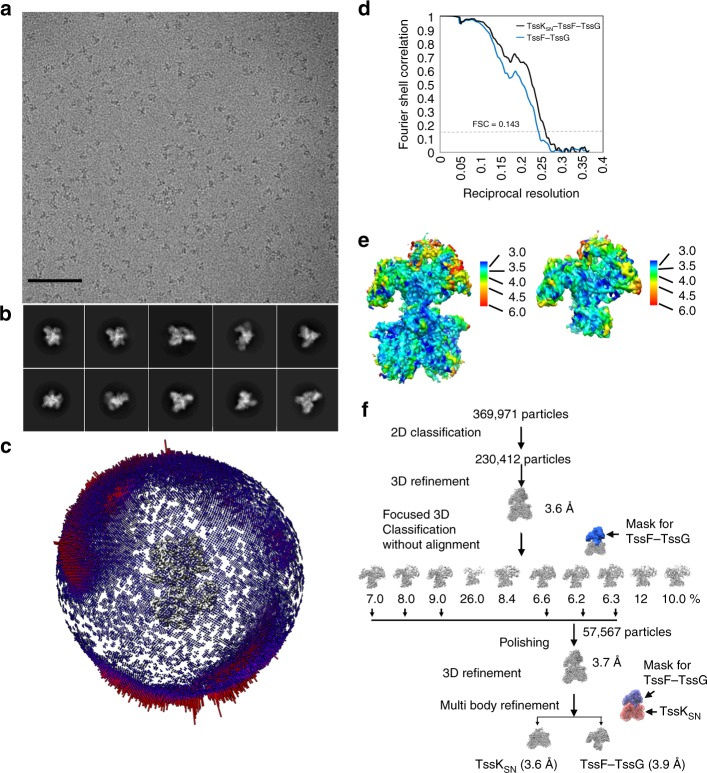
CryoEM characterization of the EAEC TssK_SN_–TssF–TssG complex. **a** Micrograph of frozen-hydrated particles adsorbed on a thin layer of carbon. Scale bar: 100 nm. **b** Reference-free 2D class averages. **c** Distribution of orientations of particle images used for the final reconstruction. **d** Gold-standard Fourier shell correlation curves for the global reconstruction (black) and the reconstruction focused on (TssF)_2_-(TssG)_1_ (blue). The 0.143 cutoff is indicated in gray. **e** Local resolution estimates of the global and TssF-TssG focused maps. **f** Flow-chart summarizing the data processing strategy employed

**Fig. 3:**
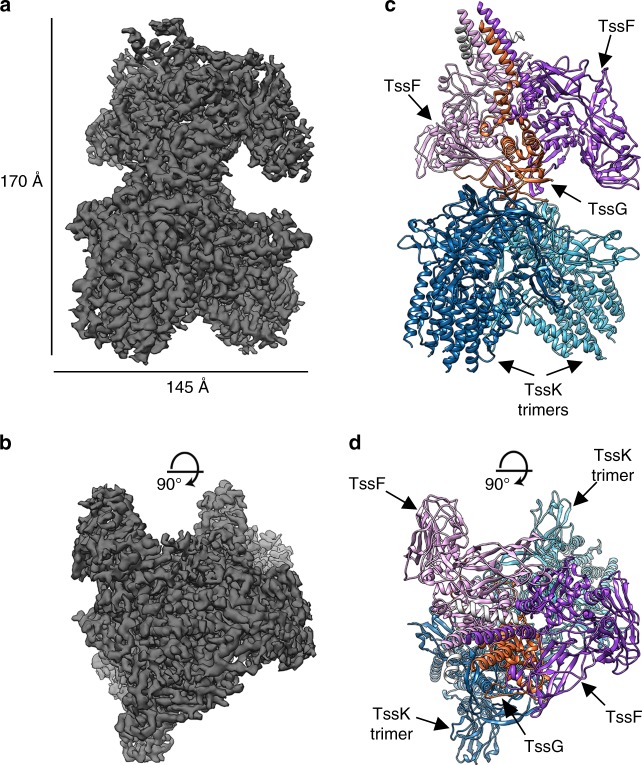
CryoEM structure of the EAEC TssK_SN_–TssF–TssG complex. **a**, **b** Two orthogonal views of the reconstruction. **c**, **d** Ribbon diagrams of the atomic model in orientations corresponding to **a**, **b**. Each subunit is colored differently. TssK: dark and light blue, TssF: purple and pink, TssG: orange, and two short unassigned segments are colored light gray

To understand the architecture of the T6SS baseplate, we overexpressed and purified an EAEC complex comprising full-lengh TssF and TssG (residues 64–366) as well as the shoulder and neck domains of TssK (TssK_SN_, residues 1–315) (Fig. 1b). We omitted from our construct the C-terminal domain of TssK (head domain), which participates to attaching the baseplate to the membrane complex, as it was previously shown to be poorly ordered and highly dynamic relative to TssK_SN_^24^. Size-exclusion chromatography indicated TssK_SN_–TssF–TssG eluted as a ~ 400 kDa complex comprising multiple copies of one or several constituting proteins (Fig. 1c, d), as reported^24,27^. Processing of the cryoEM data set revealed TssF–TssG was mobile relative to TssK and we relied on extensive 3D classification of the data, to computationally isolate a homogeneous subset of particle images^29^, as well as multi-body focused refinement with density subtraction^30^ (Fig. 2a–f). The final cryoEM map has an overall resolution of 3.7 Å (3.9 Å for the TssF–TssG focused map) and is best resolved for the region corresponding to TssK, TssG, and the C-terminal domains of TssF (Fig. 2e, Fig. 3a, b, and Table 1). The N-terminal and central domains of TssF feature weaker density owing to conformational heterogeneity (Fig. 2e and Fig. 3a, b).

**Table 1:** CryoEM data collection, refinement, and validation statistics

	TssK_SN_FG (EMDB-9341) (PDB 6N38)	TssK_SN_ (EMDB-9342)	TssFG (EMDB-9343)
*Data collection and processing*			
Magnification	36,496	36,496	36,496
Voltage (kV)	300	300	300
Electron exposure (e–/Å^2^)	40	40	40
Defocus range (μm)	0.2–4.0	0.2-4.0	0.2-4.0
Pixel size (Å)	1.37	1.37	1.37
Symmetry imposed			
Initial particle images (no.)	369,971	369,971	369,971
Final particle images (no.)	57,567	57,567	57,567
Map resolution (Å)	3.7	3.6	3.9
FSC threshold	0.143	0.143	0.143
Map resolution range (Å)			
*Refinement*			
Model resolution (Å)	3.7		
FSC threshold	0.5		
Model resolution range (Å)			
Map sharpening *B* factor (Å^2^)	−69		
Model composition			
Non-hydrogen atoms	24,934		
Protein residues	3145		
Ligands			
R.m.s. deviations			
Bond lengths (Å)	0.013		
Bond angles (°)	1.286		
Validation			
MolProbity score	0.95		
Clashscore	1.02		
Poor rotamers (%)	0.07		
Ramachandran plot			
Favored (%)	97.20		
Allowed (%)	2.67		
Disallowed (%)	0.13		

**Fig. 4:**
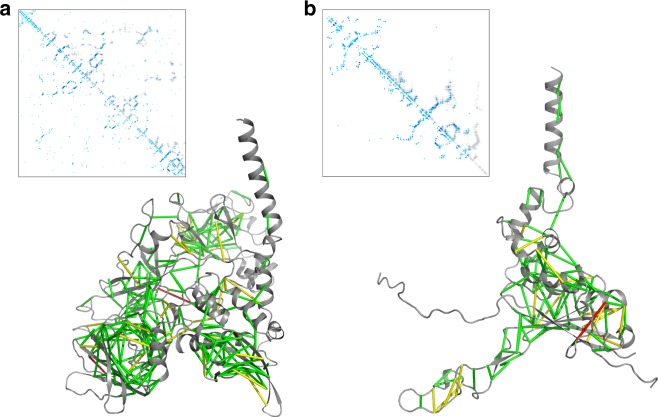
Co-evolution-predicted distance constraints for TssF and for TssG. **a**, **b** Ribbon diagrams of TssF **a** and TssG **b** showing coevolving pairs of residues with lines colored according to the distance between coevolving residues: green, Cα < 6 Å; yellow, Cα < 10 Å; red, Cα > 10 Å. The insets show contact maps from the models (light gray dots) and those predicted from co-evolution data (blue dots). The gray dots are residue contacts from 5 A (dark gray) to 10 A (light gray). The blue dots are predicted contacts, with the darker and larger dots indicating higher confidence

We obtained a complete model of the complex by combining co-evolution-based modeling^31,32^ and manual building, using Rosetta^33–37^ and Coot^38^, and docking the previously determined crystal structure of TssK^24^ into the density (Fig. 3c, d, Fig. 4a, b, and Table 1). The final model comprises TssK residues 1–315 (with a chain break between residues 220–230), TssF residues 45–587 (with a chain break between residues 393–404), as well as TssG residues 122–365 (with a chain break between residues 330–336,). The cryoEM map also contains a few unassigned densities that were modeled as polyalanine chains and which likely account for the N-terminal regions of TssF and TssG. The TssK_SN_–TssF–TssG complex forms a nine-subunit structure, comprising six copies of TssK, two copies of TssF and one copy of TssG, with dimensions of 175 Å × 145 Å × 110 Å (Fig. 3a–d).

### Architecture of the TssF–TssG heterotrimer

**Fig. 5:**
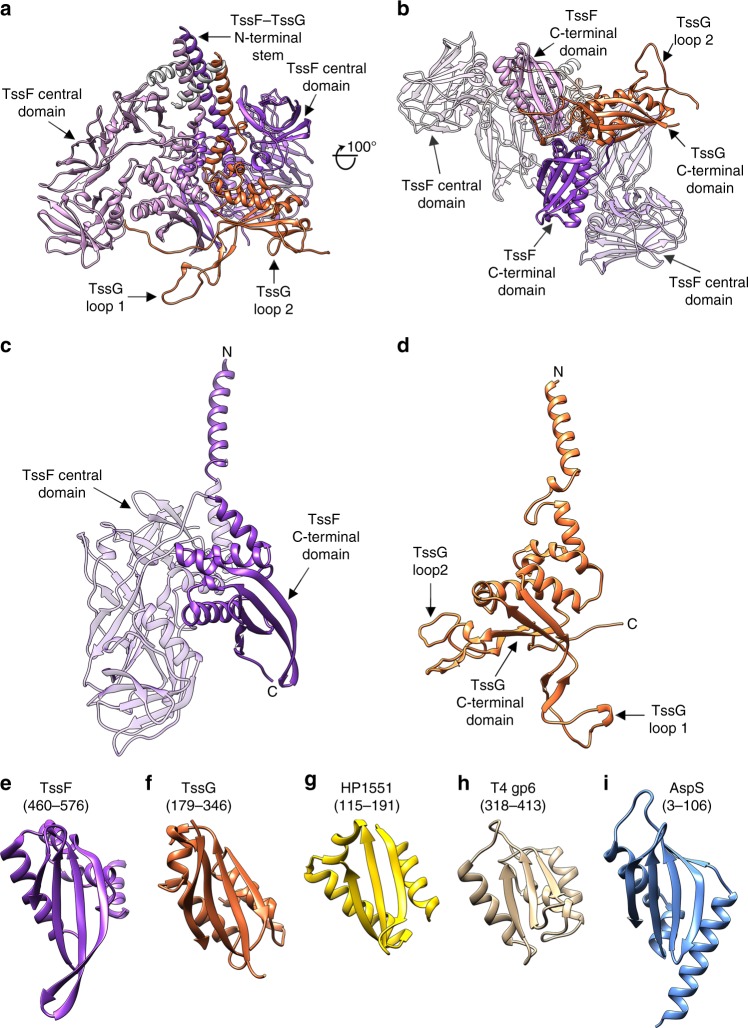
Architecture of the TssF–TssG heterotrimer. **a**, **b** Ribbon diagrams of the TssF–TssG atomic model in two quasi-orthogonal orientations. **c**, **d** A TssF protomer **c** and a TssG protomer **d** oriented similarly to each other to emphasize their structural similarity. The TssF and TssG N- and C-termini are labeled. **e**, **f** The TssF **e** and TssG **f** C-terminal domains. **g** *H. pylori* T4SS HP1451 (gold, PDB ID 2PT7, 3.9 Å r.m.s.d. over 76 Cα carbons aligned with TssF). **h** Phage T4 gp7 (tan, PDB ID 5IV5, 4.2 Å r.m.s.d. over 43 Cα carbons aligned with TssF). **i** *V. cholerae* T2SS AspS pilotin (blue, PDB ID 4FTF, 2.7 Å r.m.s.d. over 62 Cα carbons aligned with TssF). TssF and TssG are colored identically to Fig. 1 except for the TssF central domains, which are rendered semi-transparently in **b**, **c** for clarity. For **e**–**i**, the residue boundaries of the domains are shown in parentheses

TssF folds as a three-domain polypeptide comprising: (i) an α-helical N-terminus (residues 47–85); (ii) a central domain made up of three β-barrels (residues 90–457); and (iii) a C-terminal four-stranded mixed β-sheet packed against α-helices via a hydrophobic core (residues 460–587, Fig. 5a–c). TssG adopts a fold similar to TssF although the two proteins do not share detectable sequence similarity. However, only the N-terminal α-helical region (residues 122–177) and the C-terminal four-stranded mixed β-sheet supplemented with α-helices (residues 179–365) are present in TssG, which lacks an equivalent to the TssF central domain (Fig. 5a, b, d). Two extended loops, designated loop 1 and loop 2, protrude from opposite ends of the TssG β-sheet but are absent in the corresponding domain of TssF (Fig. 5c, d). The C-terminal domains of the two structures can be superimposed with an r.m.s.d of 4.5 Å over 56 aligned Cα positions (Fig. 5e, f).

The (TssF)_2_–(TssG)_1_ complex assembles as a locally pseudo-symmetrical heterotrimer with a 2:1 stoichiometry (Fig. 5a, b). The N-termini of the three modeled chains form a three-helix bundle interacting with a triangular core, which is formed upon trimerization of the C-terminal domains of TssF and TssG. The triangle is capped by the TssG loop 1 at its apex and the TssG loop 2 projects toward the periphery. The heterotrimer is highly interdigitated and buries an average surface area of 2850 Å^2^ and 2550 Å^2^ at the interface between TssG and each TssF protomer, whereas the two TssF protomers bury an average surface area of 1900 Å^2^.

The TssF and TssG C-terminal domains share remote structural similarity with the *Helicobacter pylori* HP1451 protein (Fig. 5g), which has been shown to bind to the T4SS HP0525 ATPase to regulate secretion^39^, the bacteriophage T4 baseplate proteins gp6 and gp7 (Fig. 5h) and the type II secretion system AspS pilotin (Fig. 5i). These findings reinforce the previously proposed evolutionary connection between T6SSs, tailed phages and T4SSs, which appear to share a common set of building blocks^6,11–13,17^. Our data also suggest that an ancestral duplication event and subsequent loss or addition of the TssF central domain could have led to TssG or TssF, respectively, as previously hypothesized^40^.

### Attachment of TssK to the TssF–TssG heterotrimer

The architecture of the two TssK_SN_ trimers in the TssK_SN_−TssF−TssG complex is very similar to the one observed in the TssK structure determined in complex with nanobodies nb18 and nb27 using X-ray crystallography^24^ (1.1 Å r.m.s.d. over 230 aligned Cα carbons). The TssK_SN_ trimer folds as an N-terminal β-sandwich (shoulder domain) followed by a four-helix bundle (neck domain) and resembles phage receptor-binding proteins^24^.

**Fig. 6:**
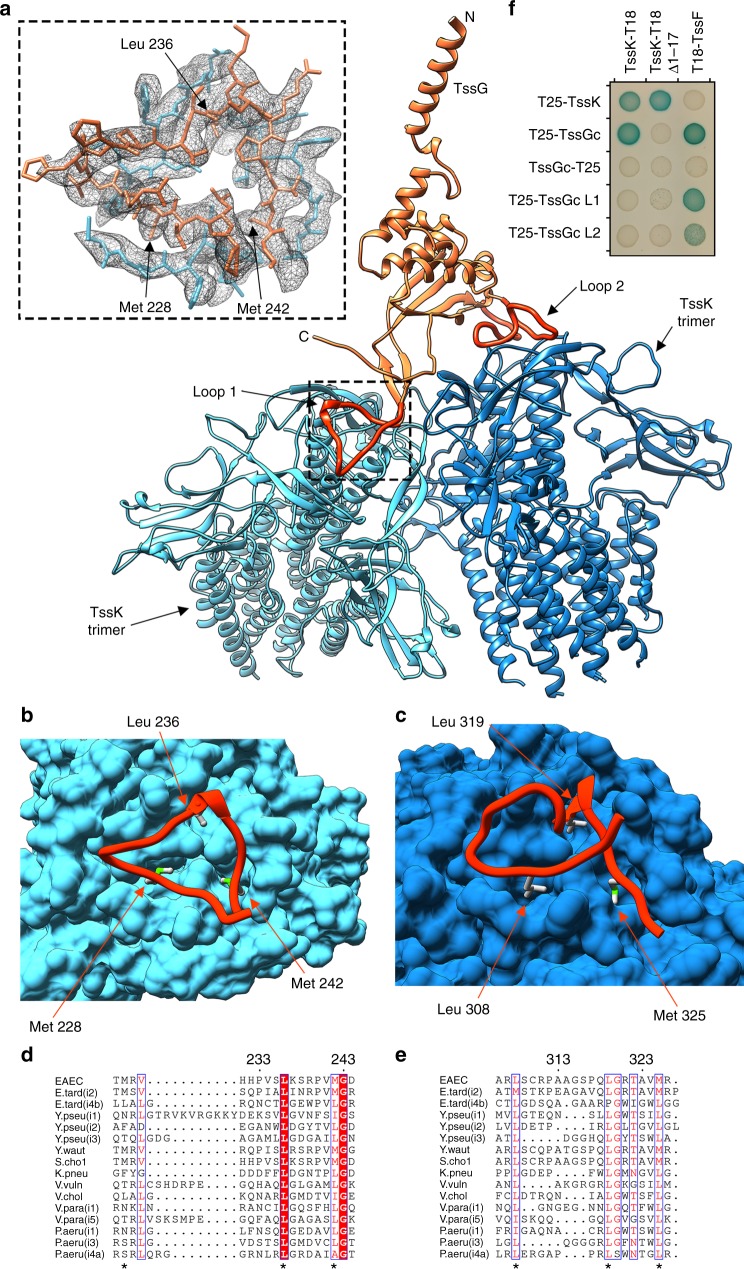
Attachment of TssK to the TssF–TssG heterotrimer. **a** Ribbon diagram showing that TssG loop 1 and loop 2 each anchor one TssK trimer to a baseplate wedge. The TssG N- and C-termini are labeled. The inset shows the atomic model built into the cryoEM density (gray mesh). **b**, **c** Zoomed-in view of the TssG loop 1-TssK **b**) and TssG loop 2-TssK **c** interactions. **d**, **e** Sequence alignment of TssG loop 1 **d** and loop 2 **e** from different bacterial species. In panels **a**, **c**, TssG is colored identically to Fig. 1 except for loop 1 and loop 2 that are rendered in orange/red and TssF is omitted for clarity. In **b**, **c**, TssK is rendered in surface representation. Hydrophobic residues that were mutated for the BACTH assay are labeled in **a**–**c** and indicated with * in d–e. EAEC: enteroaggregative *Escherichia coli*, E. tard (i2): *Edwardsiella tarda (subtype i2)*, E. tard (i4b): *Edwardsiella tarda (i4b)*, Y pseu (i1): *Yersinia pseudotuberculosis (i1)*, Y pseu (i2): *Yersinia pseudotuberculosis (i2)*, Y pseu (i3): *Yersinia pseudotuberculosis (i3)*, Y waut: *Yersinia wautersii*, S. chol: *Salmonella choleraesuis*, K. pneu: *Klebsiella pneumoniae*, V. vuln: *Vibrio vulnificus*, V. chol: *Vibrio cholerae*, V. para (i1): *Vibrio parahaemolyticus (i1)*, V. para (i5): *Vibrio parahaemolyticus (i5)*, P. aeru (i1): *Pseudomonas aeruginosa (i1)*, P. aeru (i3): *Pseudomonas aeruginosa (i3)*, P. aeru (i4a): *Pseudomonas aeruginosa (i4a)*. **f** Bacterial two-hybrid analysis of protein–protein interactions within the EAEC TssK–TssF–TssG complex. BTH101 reporter cells producing the indicated proteins or domains (TssGc: C-terminal domain of TssG, TssGc L1: C-terminal domain of TssG with the Met228Arg, Leu236Arg and Met242Arg substitutions, TssGc L2: C-terminal domain of TssG with Leu308Arg, Leu319Arg and Met325Arg substitutions, and TssK Δ1–17: TssK with deletion of the 17 *N*-terminal residues) fused to the T18 or T25 domain of the *Bordetella* adenylate cyclase were spotted on plates supplemented with IPTG and the chromogenic substrate X-Gal. Interaction between the two fusion proteins is attested by the dark blue color of the colony

Each TssK_SN_ trimer attaches to one of the two extended loops protruding from the TssG C-terminal domain. Loop 1 anchors a TssK trimer near the apex of the pseudo-threefold symmetrical (TssF)_2_–(TssG)_1_ C-terminal triangle, whereas loop 2 binds another TssK_SN_ trimer at the periphery of the triangle (Fig. 5a–d and Fig. 6a). The two TssK trimers are tilted ~ 60° relative to each other and interact together, burying an average surface area of 1000 Å^2^ at their interface. These results likely explain the observation of TssK trimers and of TssK trimers and hexamers for the EAEC and *Serratia marcescens* orthologues, respectively, as the protein forms homotrimers that interact with each other within and across wedges^28,41^. TssG interacts with excellent surface complementarity with the N-termini of the TssK trimers. Specifically, three residues from loop 1 (Met228, Leu236, and Met242) or three residues from loop 2 (Leu308, Leu319, and Met325) project into the hydrophobic cavity defined by the N-terminal 15 residues of a TssK trimer, and these interactions are further strengthened via hydrogen-bonding and salt bridges at the periphery (Fig. 6b, c). TssG loop 1 and loop 2 bury an average of 1066 Å^2^ and 1249 Å^2^ at the interface with a TssK trimer, respectively, whereas TssF weakly interacts with TssK within a baseplate wedge. Key hydrophobic residues in the TssK-binding loops are conserved (or conservatively substituted) across TssG orthologues found in distant Gram-negative bacterial species and TssG paralogues from different T6SSs (Fig. 6d, e). These findings indicate that the interaction between TssG and TssK are most likely a common feature of T6SSs. Binding of TssK to TssG is reminiscent of the attachment mode observed for receptor-binding proteins to phage baseplates, such as in the cases of TP901–1^42^ or p2^43^ although the molecular details of the interactions are distinct.

### TssK_SN_–TssF–TssG interactions

To validate the interactions detected in our cryoEM reconstruction, we used a bacterial two-hybrid (BACTH) assay^44^. Our structure predicts that TssK protomers interact, as the protein forms homotrimers that contact each other. Indeed, BACTH confirmed the self-association of TssK (Fig. 6f and Supplementary Figure 1). We next confirmed that the C-terminal domain of TssG acts as an adaptor for the TssK_SN_–TssF–TssG complex by interacting with TssK and with TssF (Fig. 6f and Supplementary Figure 1), as observed in the atomic model. We interpret the lack of TssG_C_–T25 interactions with TssF and with TssK as a result of steric hindrance mediated by fusing the adenylate cyclase T25 domain at the C-terminus of the TssG C-terminal domain. This interpretation agrees with our structural data, with the detection of T25–TssG_C_ (T25 fused at the N-terminus of the TssG C-terminal domain) interactions with TssF and with TssK, and with a previous study using a comparable experimental setup^26^.

Our structure also indicates that the TssG loop 1 and loop 2 each mediate contacts with the N-terminal region of one TssK trimer. BACTH experiments support these findings since (i) removal of the TssK N-terminal 17 residues or (ii) substitution with arginine of the aforementioned three hydrophobic residues within the TssG loop 1 (Met228Arg, Leu236Arg, Met242Arg) or loop 2 (Leu308Arg, Leu319Arg, Met325Arg) abrogated binding between TssG and TssK without significantly affecting the interactions between TssG and TssF (Fig. 6f and Supplementary Figure 1). In summary, the outcomes of the BACTH experiments validated our atomic model and also agreed with and extend previously reported BACTH data^24,26^.

### Architecture of the baseplate

We generated a model of the T6SS baseplate putatively corresponding to the conformational state before sheath contraction using our structure of the EAEC TssK_SN_–TssF–TssG complex and the previously determined cryoEM reconstruction of the non-contractile sheath mutant of the *Vibrio cholerae* T6SS baseplate/needle at 8 Å resolution^45^.

**Fig. 7:**
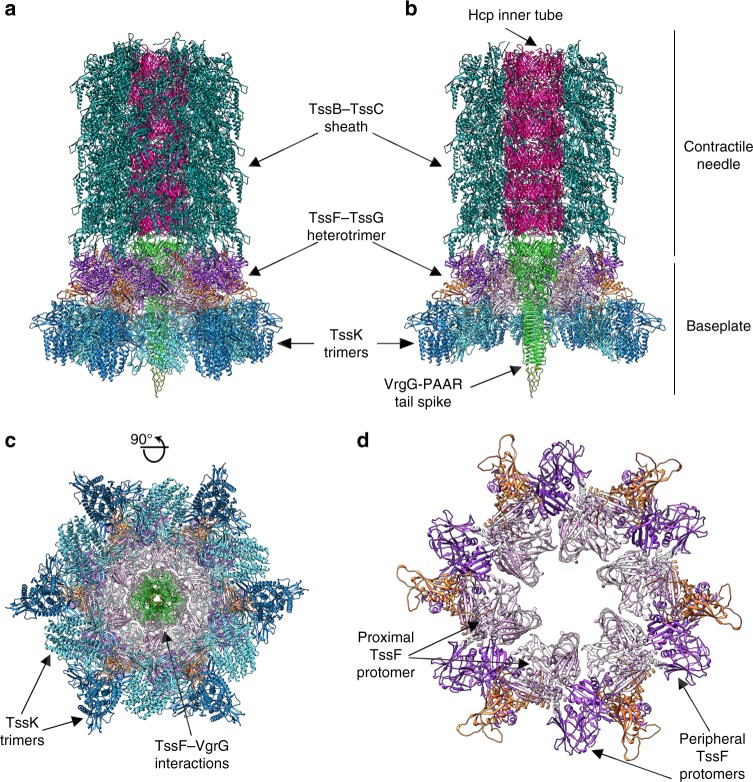
Model of the T6SS baseplate and needle complex architecture before sheath contraction. **a** A model was obtained by rigid-body docking subcomplex structures into the single particle cryoEM reconstruction of the non-contractile sheath mutant of the *Vibrio Cholerae* T6SS baseplate/needle at 8 Å resolution (EMD 3879)^45^. **b** Cut-away view of the model shown in **a**. **c** Orthogonal view of the model shown in **a**. **d** Circularization of the (TssF)_2_-(TssG)_1_ complex in the context of the T6SS baseplate. TssK, TssF, TssG are colored identically to Fig. 1; TssB–TssC: teal (PDB ID 3j9g)^19^; Hcp: magenta (PDB 5OJQ); VgrG-PAAR: green-olive (PDB ID 4MTK and 4JIV)^9^

The TssK–TssF–TssG complex, along with TssE, constitute a wedge and six such wedges are circularized in a baseplate comprising 36 copies of TssK, 12 copies of TssF, 6 copies of TssG and presumably 6 copies of TssE^27,40^ (Fig. 7a–c and Supplementary Figure 2a–g). Rigid-body docking the TssK_SN_–TssF–TssG structure into the baseplate map shows the architecture of the recombinant complex recapitulates its overall organization in the context of a fully assembled T6SS apparatus. Although the resolution of the baseplate map is limited, we could nevertheless notice local tertiary and quaternary structural differences likely corresponding to conformational switching upon assembly, as reported for the bacteriophage major tail protein^13^. As the TssK region features weak density in the baseplate reconstruction, owing to mobility relative to the rest of the baseplate and/or substoichiometric incorporation of TssK, we applied a low-pass filter at 20 Å resolution to enhance map resolvability. This procedure confirmed each wedge attaches two TssK trimers to the baseplate (Supplementary Figure 2a–g), in agreement with the TssK_SN_–TssF–TssG structure reported here. The apparent conformational heterogeneity of TssK further reinforces the similarity between phage and T6SS baseplates since receptor-binding proteins or TssK trimers are highly dynamic and this could be important for their functions^42^. The central domain of the proximal TssF protomer from each wedge contacts the VgrG oligosaccharide/oligonucleotide (OB)-fold barrel such as the latter domain is surrounded by two TssF protomers from two different wedges. VgrG thereby ensures the transition between its intrinsic threefold symmetry and the sixfold symmetry of the baseplate and needle, similarly to the phage T4 gp27 protein^15,27,46^ or the Tal protein of phages TP901–1^42^ and p2^43^. Lateral stabilization of the baseplate is provided by inter-wedge TssF–TssF, TssK–TssK, and TssF–TssK interactions (Fig. 7a–d). Most of the inter-wedge TssF–TssF interactions involve contacts between a proximal TssF protomer of the wedge i (through its central and C-terminal domains) and a peripheral TssF protomer of the wedge i + 1 (through its C-terminal domain, Fig. 7d).

Our model also suggests TssK–TssF–TssG complexes are interacting with the proximal TssB–TssC sheath ring, in agreement with previous BACTH studies^23,26^. On one hand, the (TssF)_2_–(TssG)_1_ heterotrimer N-terminal stem contacts TssC. On the other hand, the N-terminal helix of TssC and the C-terminal helix of TssB protrudes in direction of the same (TssF)_2_–(TssG)_1_ heterotrimer to interact with the small β-barrel located within the TssF central domain. The interactions between TssB/TssC and TssF are mediated by the TssF protomers radiating at the periphery of the baseplate whereas the TssF protomers protruding toward the center of the baseplate bind to VgrG. TssE, which is absent in the model presented here, is expected to interact with the (TssF)_2_–(TssG)_1_ heterotrimer N-terminal stem and with the TssB/TssC sheath, based on similarity with the phage T4 baseplate structure^11,27,46^.

## Discussion

The results presented here further our knowledge about the T6SS baseplate architecture and assembly pathway. Hexamerization of the (TssK)_6_–(TssF)_2_–(TssG)_1_–(TssE)_1_ wedge leads to formation of the baseplate complex around a VgrG trimer bound to a PAAR-repeat protein at its distal extremity^45^. As we could not detect baseplate-like complexes upon overexpression and purification of TssK_SN_–TssF–TssG (even at high concentration), we propose VgrG nucleates the assembly of individual baseplate wedges, as previously suggested^26^. We expect that docking of the six proximal TssF protomers, through their central domain, near one of the three VgrG OB-fold domains initiates circularization. Once adjacent baseplate wedges are hexamerized and surround the central VgrG trimer, the wedges are sealed via proximal/distal TssF interactions. In the case of EAEC, TssA, TssM, and TssL participate to docking of the baseplate to the cytoplasmic side of the inner membrane through interactions formed by (i) the TssK head domain with the cytoplasmic domains of TssL and of TssM, (ii) the N-terminal domain of TssA with TssE and TssK, (iii) the C-terminal domain of TssA with VgrG, and (iv) TssG with the cytoplasmic domain of TssM^23,24,26,47^.

Once assembly of the baseplate is completed, the tail tube and sheath polymerize, either sequentially or simultaneously, via addition of hexameric Hcp rings and of TssB–TssC rings or rows at the growing extremity^48^. Stabilization of the TssB–TssC ring directly contacting the baseplate occurs (i) through binding of TssC to the (TssF)_2_–(TssG)_1_ N-terminal helical stem, (ii) attachment of the peripheral TssF central domain to the C- and N-termini of TssB and TssC, respectively, and (iii) putatively via interactions of TssE with TssB–TssC. Additional interactions between a hexameric Hcp ring and the pseudo-six-fold symmetrical VgrG trimer participate to initiation of tube formation^23^.

The (TssF)_2_–(TssG)_1_ heterotrimer assembles with the same stoichiometry as the T4 (gp6)_2_–(gp7)_1_ heterotrimer and the four proteins appear to share a distant ancestor. Specifically, the (TssF)_2_–(TssG)_1_ C-terminal domains form a pseudo-3-fold symmetrical triangle, which resembles the T4 (gp6)_2_–(gp7)_1_ trifurcation domain recently described^27,46^ and the domains involved all adopt a similar fold. Moreover, both (TssF)_2_–(TssG)_1_ and (gp6)_2_–(gp7)_1_ form a three-helix bundle arranged roughly perpendicularly with respect to the triangle motif. This similarity is strengthened by the observation that the (TssF)_2_–(TssG)_1_ heterotrimer participate to circularizing the baseplate, recruiting TssK (which is structurally and to some degree functionally related to phage receptor-binding proteins) and interacting with the central spike and the tail sheath, similarly to T4 (gp6)_2_–(gp7)_1_^27,40^. Circularization of the T6SS and the T4 baseplates, however, is mediated by distinct types of interactions, as inter-wedge contacts occur between C-terminal triangular-shaped cores through proximal and peripheral TssF protomers or between proximal and peripheral gp6 C-terminal domains (which are distinct from the trifurcation domains), respectively. Overall, the T6SS baseplate appears to be related to the baseplate of contractile bacteriophages as (TssF)_2_–(TssG)_1_ shares structural, topological, and functional traits with T4 gp6/gp7 and orthologous proteins found in other contractile phages^27,40,49^. The baseplate of some non-contractile phages, however, rely on a single unrelated protein forming quasi-equivalent trimers, such as TP901–1 BppU^42^, to perform the same function.

## Methods

### Plasmid design and protein expression and purification

The plasmid for expression of the TssK_SN_–TssF–TssG complex (pCDF–TssK_SN_6His–TssF–TssG) comprises three open-reading frames encoding TssK_SN_ (residues 1–315) with a C-terminal His_6_-tag, full-length TssF, and TssG (residues 64–366) under the control of a single T7 promoter^24^. *Escherichia coli* BL21DE3 (Novagen) cells bearing pCDF-TssK_SN_6His–TssF–TssG were grown at 37 °C in Luria-Bertani (LB) supplemented with 50 µg/ml streptomycin. Protein expression was induced with 0.5 mM isopropyl β-d-1-thiogalactopyranoside (IPTG) for 16 h at 18 °C. Cells were harvested, resuspended in Tris-HCl 20 mM pH 8.0, NaCl 150 mM with lysozyme and lysed using a French press. Soluble proteins were separated from insoluble material by centrifugation 30 min at 20,000 g. The TssK_SN_–TssF–TssG complex was purified using Ni-NTA affinity chromatography and eluted with 300 mM imidazole. Subsequently, it was further purified by gel-filtration chromatography using a Superose 6 10/300 GL column (GE Life Sciences) equilibrated in 20 mM Tris-HCl (pH 7.8), 150 mM NaCl, 5% Glycerol, and 0.5 mM TCEP. The purity of the sample was assessed by Coomassie-stained sodium dodecyl sulfate–polyacrylamide gel electrophoresis.

### CryoEM data acquisition and processing

Three microliters of the purified TssK_SN_–TssF–TssG complex (0.12 mg/ml) in 20 mM Tris-HCl pH 7.8 and 150 mM NaCl was applied to glow discharged C-flat holey carbon grids covered with a thin layer of carbon. Grids were then plunge-frozen in liquid ethane with an FEI MK4 Vitrobot with a 3 s blot time. The chamber was maintained at 20 °C and 100% humidity during the blotting process. Data were collected with the Leginon data collection software^50^ on an FEI Titan Krios electron microscope operated at 300 kV and equipped with an energy filter (slit width of 20 eV), and a direct electron detector Gatan K2 Summit. The dose rate was adjusted to eight counts per pixel per second, and each movie was acquired in super-resolution counting mode fractionated in 50 frames of 200 ms. A total of 2601 micrographs were collected with a defocus range between 0.2 and 4.0 μm. Particles were automatically selected using Dog Picker^51^ within the Appion pipeline^52^. Movie frame alignment was carried out with dose weighting using MotionCorr2^53^. Defocus parameters were estimated with GCTF^54^. An initial model was obtained using cryoSPARC^55^ and all 2D and 3D classifications and refinements were performed using RELION 2.1. Subsequently, Relion3.0beta was used to refine per-particle defocus values before reclassification of the data focusing on the TssF–TssG C-terminal domain. Finally, multi-body refinement^30^ was used with signal subtraction to produce a map focused on the two TssK trimers and a map focused on the (TssF)_2_–(TssG)_1_ heterotrimer. The reported resolution is based on the gold-standard FSC = 0.143 criterion^56,57^ and Fourier shell correlation curves were corrected for the effects of soft masking by high-resolution noise substitution^58^. Local resolution estimation was performed using blocres^59^.

### Model building and refinement

TssK was modeled based on the available X-ray crystal structures^24^, whereas TssF and TssG were modeled de novo by initially building a backbone trace of their C-terminal domains into a preliminary 4.2 Å resolution reconstruction. Modeling of TssF and TssG relied on both the global map and the focused map obtained using multi-body refinement. Co-evolution derived constraints provide a way of inferring structural information given large families of evolutionarily related sequences of a protein. By identifying residues that frequently mutate together, structural information may be inferred. The strength of this “co-evolution” is strongly predictive of residue–residue contacts in the 3D structure of the protein^31,32,36,60^. For both TssF and TssG, there was sufficient sequence data to perform co-evolution analysis, and models were built using Rosetta guided by this co-evolution data^32^. These models were built by domain (three domains for TssF and two domains for TssG), and the C-terminal domain models were converged and placed into density with the help of the aforementioned backbone trace (a long insertion corresponding to TssG loop 1 allowed discrimination of TssF from TssG density). Several long insertions that poorly converged using the co-evolution information alone were rebuilt into density using RosettaES^37^. Subsequent modeling of the TssG coordinates was carried out using a combination of Coot^38^ and Rosetta^34,35^ refinement, with the final model refined with Rosetta. The rest of the TssF structure (residues 20–459) was derived from the Rosetta co-evolution model and fit into density before rebuilding and refinement using a combination of Coot^38^ and Rosetta^34,35^. Building and refinement was carried out using the TssK and TssF–TssG focused maps before performing a final refinement round in the TssK–TssF–TssG map. The TssF and TssG atomic models agree with 247 out of 250 and 105 out of 106 co-evolution constraints, respectively. Interaction surface area was calculated using PISA^61^. All figures were generated with UCSF Chimera^62^ and UCSF ChimeraX^63^.

### Bacterial two-hybrid assays and western blotting

*E. coli* BTH101 cells were co-transformed with plasmids encoding the T18 and T25 fragments of *Bordetella pertussis* adenylate cyclase fused to the proteins of interest. Stationary phase cells were normalized to OD_600_ 0.5 and plated on LB agar containing 80 mg/mL X-gal, 0.5 mM IPTG, 50 mg/mL kanamycin and 150 mg/mL carbenicillin and grown for 24 h at 30 °C. pKT25 and pKNT25 constructs added T25 to the N or C-terminus, respectively, and the transformed cells were grown in the presence of 50 mg/mL kanamycin. pUC18 and pUC18C constructs added T18 to the C or N-terminus, respectively, and the transformed cells were grown in the presence of 150 mg/mL carbenicillin. Cloning was performed using Gibson assembly. Western blotting was performed using a mouse anti-CyaA (1:5000, Santa Cruz Biotechnologies #: SC-13582, Lot: C2715) and detected with an anti-mouse horseradish peroxidase-conjugated antibodies (1:5000, Sigma). Western blots were developed using chemiluminescent substrate (SuperSignal West Pico Substrate, Thermo Scientific) and imaged with an Azure c500 (Azure Biosystems). The leucine zipper interactions of the yeast protein GCN4 served as a positive control.

## Supplementary information

Supplementary Information

Reporting Summary

## Data Availability

The sharpened and unsharpened cryoEM reconstruction of TssK_SN_–TssF–TssG, the focused reconstructions of TssK and TssF–TssG, and atomic model have been deposited in the Electron Microscopy Data Bank and the Protein Data Bank with accession codes EMD-9341 (TssK_SN_–TssF–TssG map), EMD-9342 (TssK focused map), EMD-9343 (TssF–TssG focused map), and PDB 6N38. Other data are available from the corresponding author upon reasonable request.
