# Supplementary material for: Structure of the type VI secretion system TssK–TssF–TssG baseplate subcomplex revealed by cryo-electron microscopy
Source: Nat Commun. 2018 Dec 19;9:5385. doi: 10.1038/s41467-018-07796-5 (PMC6300606; doi:10.1038/s41467-018-07796-5)
Supplement: Supplementary file 1 — Supplementary Information [file 41467_2018_7796_MOESM1_ESM.pdf]

Structure of the type VI secretion system TssK-TssF-TssG baseplate subcomplex revealed by cryo-electron microscopy

Supplementary Information

Young-Jun P *et al*[1]

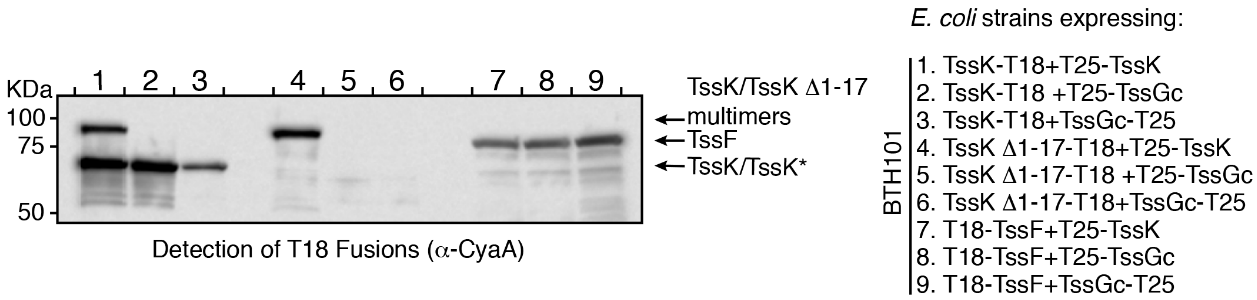

Supplementary Figure 1. Western blot of T18-fused expression constructs utilized in BATCH.

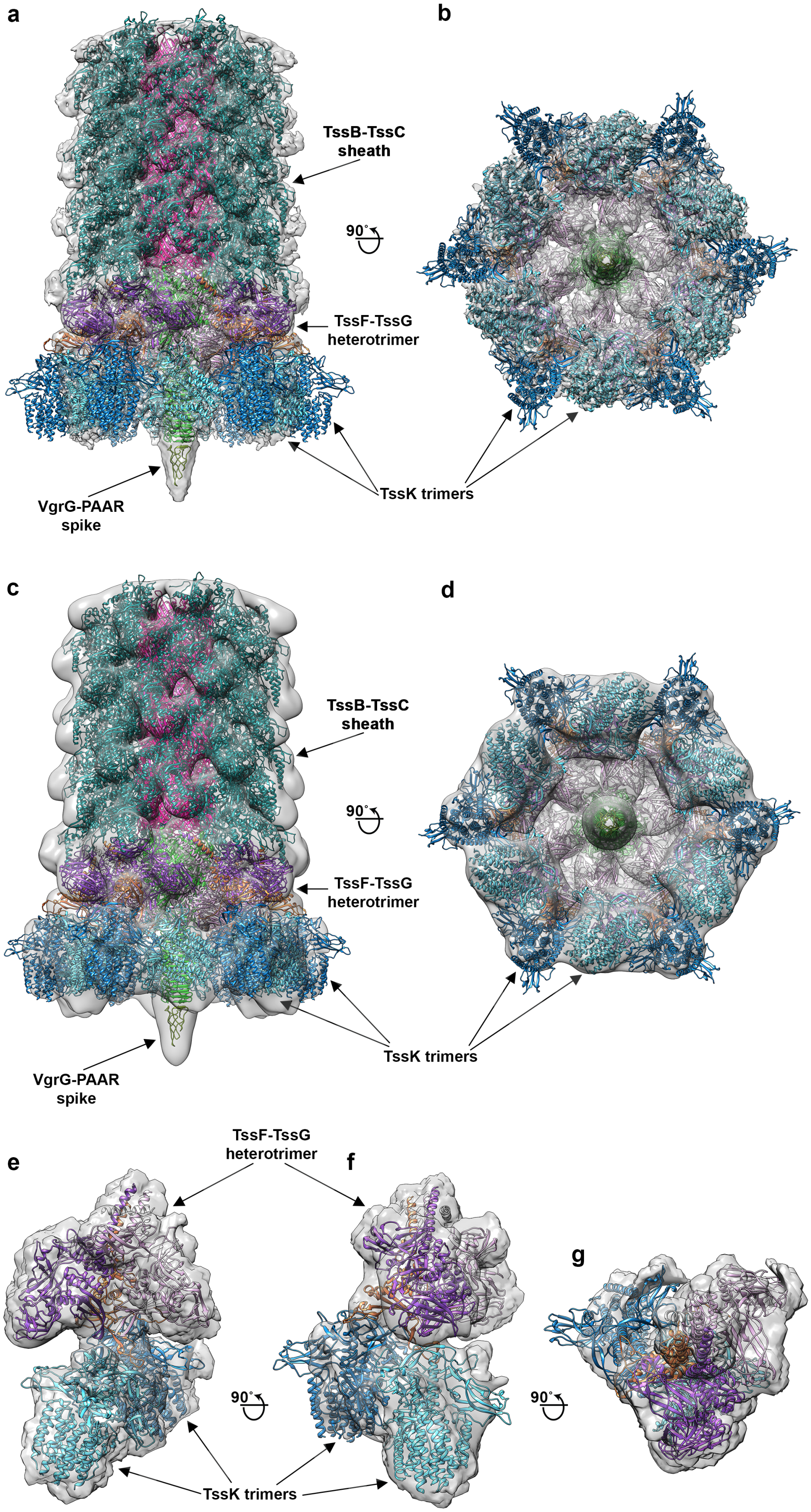

a
b
TssB-TssC
sheath
90°
TssF-TssG
heterotrimer
VgrG-PAAR
spike
TssK trimers
c
d
TssB-TssC
sheath
90°
TssF-TssG
heterotrimer
VgrG-PAAR
spike
TssK trimers
TssF-TssG
heterotrimer
e
f
g
90°
90°
TssK trimers

Supplementary Figure 2. Supramolecular architecture of the T6SS baseplate and needle complex before sheath contraction. a-b. Two orthogonal views of the model obtained by rigid-body docking subcomplex structures into the single particle cryoEM reconstruction of the non-contractile sheath mutant of the *Vibrio Cholera* T6SS baseplate/needle at 8 Å resolution (EMD 3879)[45]. TssK trimers are poorly resolved due to conformational heterogeneity and/or substoichiometric incorporation. **c-d.** Identical renderings than the ones shown in panels (c-d), after low-pass filtering the T6SS baseplate/needle map to 20 Å resolution, showing resolved density for TssK. **e-g.** Ribbon diagrams in orthogonal orientations of the $TssK_{SN}$-TssF-TssG structure determined in this manuscript fitted in the T6SS baseplate/needle map low-pass filtered to 20 Å. $TssK_{SN}$-TssF-TssG: blue-purple-pink, with two short unassigned segments colored light grey; TssB-TssC: teal (PDB ID 3j9g)[19]; Hcp: magenta (PDB 5OJQ); VgrG-PAAR: green-olive (PDB ID 4MTK and 4JIV)[9].

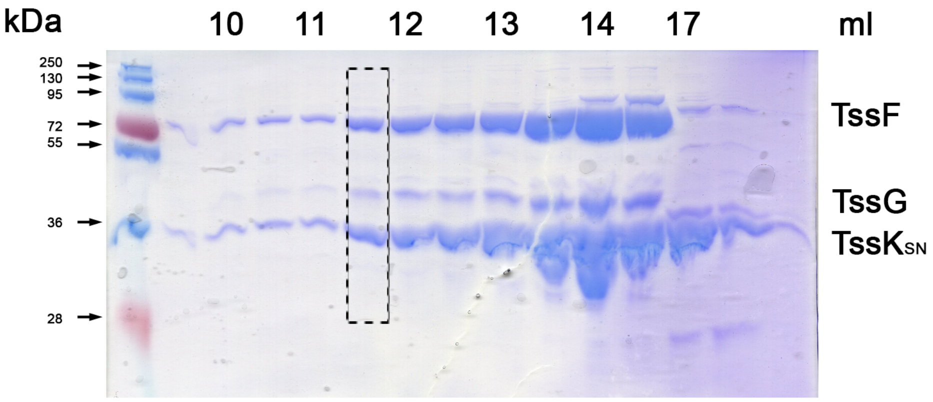

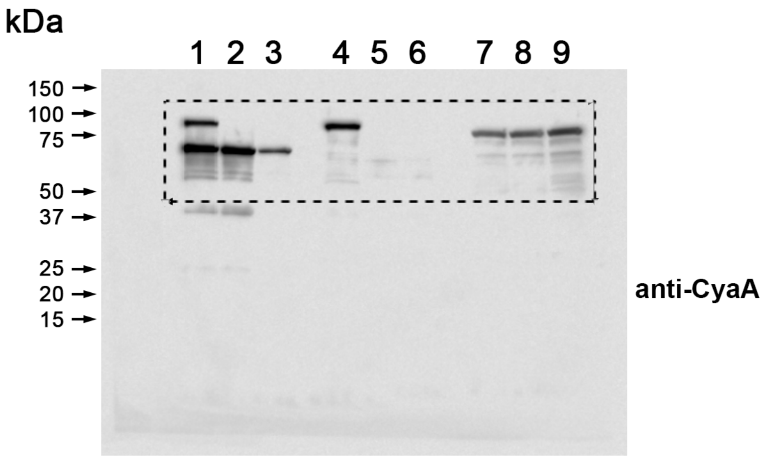

Supplementary Figure 3. Uncropped blots and gels.
